# Extension of Lander-Waterman theory for sequencing filtered DNA libraries

**DOI:** 10.1186/1471-2105-6-245

**Published:** 2005-10-10

**Authors:** Michael C Wendl, W Brad Barbazuk

**Affiliations:** 1 Genome Sequencing Center, Washington University, St. Louis MO 63108, USA; 2 Donald Danforth Plant Science Center, St. Louis MO 63132, USA

## Abstract

**Background:**

The degree to which conventional DNA sequencing techniques will be successful for highly repetitive genomes is unclear. Investigators are therefore considering various filtering methods to select against high-copy sequence in DNA clone libraries. The standard model for random sequencing, Lander-Waterman theory, does not account for two important issues in such libraries, discontinuities and position-based sampling biases (the so-called "edge effect"). We report an extension of the theory for analyzing such configurations.

**Results:**

The edge effect cannot be neglected in most cases. Specifically, rates of coverage and gap reduction are appreciably lower than those for conventional libraries, as predicted by standard theory. Performance decreases as read length increases relative to island size. Although opposite of what happens in a conventional library, this apparent paradox is readily explained in terms of the edge effect. The model agrees well with prototype gene-tagging experiments for *Zea mays *and *Sorghum bicolor*. Moreover, the associated density function suggests well-defined probabilistic milestones for the number of reads necessary to capture a given fraction of the gene space. An exception for applying standard theory arises if sequence redundancy is less than about 1-fold. Here, evolution of the random quantities is independent of library gaps and edge effects. This observation effectively validates the practice of using standard theory to estimate the genic enrichment of a library based on light shotgun sequencing.

**Conclusion:**

Coverage performance using a filtered library is significantly lower than that for an equivalent-sized conventional library, suggesting that directed methods may be more critical for the former. The proposed model should be useful for analyzing future projects.

## Background

Over the last few decades, DNA sequencing has firmly established its role in the broader enterprises of scientific and medical research. Enabled by ongoing development and refinement of laboratory techniques, instruments, and software, investigators are now studying a wide array of genomes at a level of sophistication not before possible. While a number of sequencing approaches have been devised, experience indicates that the efficacy of any particular one depends strongly upon the context of the target sequence. For instance, the whole genome shotgun (WGS) procedure has proved especially suited to microbes [1]. Conversely, mammalian projects are being completed using large-insert mapped clones, which are better able to resolve long-range assembly issues related to DNA repeats [2].

Repetitive sequences are especially abundant in many of the economically and agriculturally important plant species. For example, the maize genome (*Zea mays*) is comparable in size to the human genome, yet up to 80% of it consists of retroelements [3,4]. The degree to which established sequencing techniques will be successful for such cases is not clear [5]. Two notable methods have been proposed to address high-repeat projects. Both seek to filter out repetitive regions, leaving primarily low-copy "islands" to be amplified in a DNA library. Methyl filtering excludes repetitive elements based on their elevated levels of cytosine methylation [6]. Conversely, high-Cot purification preferentially selects low-copy genic regions based upon characteristic re-association rates [7,8]. In this context, it can be considered a form of normalizing procedure. Methyl filtering appears to be compatible only with plant genomes [9], while Cot selection can be applied broadly.

**Figure 1:**
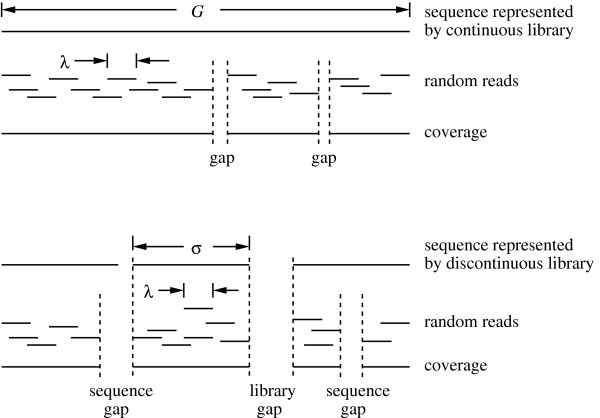
Schematic of the covering process for the conventional continuous library (top) versus the filtered discontinuous library (bottom).

Genomic projects are generally guided by probabilistic models of the underlying random processes. The seminal work of Lander and Waterman [10] has long served as the theoretical foundation for standard fingerprint mapping and shotgun sequencing methods. Although not strictly correct, the coverage model first used by Clarke and Carbon [11] is also treated as a *de facto *part of Lander-Waterman (LW) theory. These formulations are predicated upon an infinitely long genome, whose sequence is completely represented in the form of a non-biased clone library. In mathematical terms, these clones and their resulting sequence reads are taken to be independently and identically distributed (IID). The LW model allows one to estimate parameters of interest, e.g. sequence coverage and the number of gaps, as functions of the number of reads processed (Fig. 1, top).

Filtered libraries are, however, an *incomplete *representation of the target sequence. Specifically, they are punctuated by fixed gaps of unknown size (Fig. 1, bottom). When using the filtering schemes mentioned above, the number of such gaps is expected to be large and this introduces additional modeling issues. Consider, for example, a *de novo *assembly. Without independent linking information, it is not strictly possible to distinguish between the fixed gaps native to the library and the sequence gaps that evolve stochastically as a part of the coverage process. Under ideal conditions, library gaps would clearly manifest themselves only in the limit of an infinite number of clones, because all sequence gaps should vanish.

So-called "edge effects" must also be considered for filtered libraries. Demonstrating this phenomenon is a matter of simple probability. Suppose a genic island of size *σ *is being covered by random sequence reads of length *λ*, where *λ *≪*σ*. The terminal base position has only about a 1/*σ *chance of being covered by any individual read, while the associated probability for an interior base position (far from the terminus) is roughly *λ/σ*. Such differences can be regarded as a form of position-based sampling bias because preference for coverage is clearly shifted toward the interior island regions. The fraction of an island affected in this way can have significant implications on the evolution of coverage and gaps.

Here, we report an extension to the standard LW theory for filtered library configurations. It describes not only the analogs of established LW parameters, but also several new quantities of interest that arise as a consequence of the fragmented nature of the library. Preliminary experimental results have been favorable [12-14], suggesting that filtering procedures will be applied on a broader scale to the most recalcitrant genomes. For such projects, investigators must currently rely on a casual, but unproven adaptation of LW theory. Here, all genic islands are artificially concatenated into a single "super-island" and the size of this island is taken as the effective genome size. We will refer to this idealization as the Lander-Waterman Super Island (LWSI) model. Because this representation neglects library gaps and the associated edge effects, the degree to which it is applicable to actual projects is not known.

## Results and discussion

### The mathematical model

A gap consists of any genomic region following a read that is not manifested as coverage. Two types of gaps arise under this definition. If the uncovered region is represented in the library it is called a "sequence gap", otherwise it is called a "library gap" (Fig. 1, bottom). The numbers of sequence and library gaps are denoted by the random variables *S *and *L*, respectively. We also define the following random variables: *C *is the number of bases covered, *I *is the number of islands hit by at least one sequence read, and *R *denotes the number of reads hitting a particular island. (Table 1 summarizes the mathematical notation used in the model.)

**Table 1:** Mathematical notation

symbol	type or formula	meaning
*C*	random variable	number of bases covered
*I*	random variable	number of islands hit by at least one read
*L*	random variable	number of library gaps
*R*	random variable	number of reads hitting a particular island
*S*	random variable	number of sequence gaps
*λ*	parameter	length of a sequencing read
*σ*	parameter	size of a filtered island
*i*	parameter	number of filtered islands
*n*	parameter	number of sequencing reads processed
*π*	*σ *- *λ *+ 1	number of placements for a read on an island
Π	*i*(*σ *- *λ *+ 1)	number of possible placements over whole target

Let the filtered library consist of *i *islands, each of which is *σ *base-pairs in size. Reads are taken to be of length *λ *base-pairs and are assumed to be IID, as in the standard LW model. Read length may include a reduction factor to account for the number of bases effectively lost in detecting overlap with another read [10]. We presume, as an upper bound, that read length does not exceed island length, i.e. *λ *≤ *σ*

There are *π *= *σ *- *λ *+ 1 possible placements of a read on each island, and consequently, Π = *iπ *total placements within the library. Assuming *n *reads have been processed, the expected values of the random variables are given by the following theorems.

**Theorem 1 (library gaps)**. *The expected number of library gaps is*

*E*〈*L*〉 = *i*(1 - *e*^*-n*/Π^),

*where e *≈ 2.71828 *is Euler's number*.

**Theorem 2 (sequence gaps). ***The expected number of sequence gaps when λ *≤ (*σ *+ 1)/2 *is*



*where n*/Π *is constrained according to Lemma 2, ρ = nλ*/Π, *and δ = σ *- 2(*λ *- 1).

**Theorem 3 (coverage). ***The expected number of bases represented by the library that are covered by at least one sequence read is*



*where λ ≤ σ*/2.

**Theorem 4 (reads per island). ***The number of sequence reads placed on a specific island follows a Poisson distribution with an average value (rate) of E*〈*R*〉= *n/i. In particular, the probability that the island is not hit by any reads is *exp(-*n/i*).

**Theorem 5 (number of islands hit). ***The distribution of the number of genic islands hit by one or more sequence reads is*



*where μ *= *i *exp(-*n/i*) *and the expected value is*

*E*〈*I*〉 = *i*(1 - *e*^*-n/i*^).

Theorems 2 and 3 have been derived according to parameters of current biological interest (Lemma 2). They also adhere to their respectively stated, but less-restrictive mathematical conditions. However, it is straightforward to modify them when λ is larger relative to *σ *(see Methods).

These results enable one to probabilistically characterize the shotgun sequencing process for filtered DNA libraries in much the same way that standard LW theory is used for conventional libraries. Filtering is expected to play a significant role for the most difficult, repeat-laden genomes, where cost and assembly issues may limit the success of conventional techniques.

Investigators have had to rely on a rudimentary adaptation of LW theory, whereby the fragmented library is modeled as a single "super-island" [7,12,14]. Here, there are *i σ *- *λ *+ 1 ≈ *i σ *possibilities for placing clones of length *λ*. In actuality, significantly fewer placements exist, *i*(*σ - λ *+ 1), owing to the discontinuities between islands. Some statistics will be dramatically skewed as a consequence, for example the expected contig size will not converge to the correct value of *σ*. Accuracy of other quantities is not clear. Also, there is no provision to estimate island-specific statistics, such as the number of islands hit by at least one read. The idealized LWSI model is correct only for the special case *i *= 1, although errors for some of the variables will be minimal if *λ/σ *is sufficiently small.

Here, we examine the sequencing process over a range of parameters to discern the general trends that one should be aware of. We also characterize some of the practical applications relevant to filtered libraries and assess the applicability of the "super island" (LWSI) assumption. Our model can readily be applied to specific projects, as well.

### Coverage characteristics

Maize can be taken as a representative high-repeat genome. Whitelaw et al. [12] describe shotgun sequencing from filtered libraries. They report an average read length of 719 bases and 50 base minimal overlap, so that the effective read length is *λ *≈ 669 bp. Genome size and repeat content are taken as 2.7 Gb and 80%, respectively [15]. If, for the moment, we assume perfect filtering, the resulting library would comprise about 540 Mb of DNA sequence.

Island size, unlike read length, cannot readily be characterized *a priori*. Maize genes are thought to reside predominantly in small, roughly 3 kb regions of unmethylated DNA, which are surrounded by tracts of 20–200 kb highly methylated, high-copy sequence [3,16,17]. Thus, the maize gene space appears to be well-dispersed across the physical genome with most genes being distinctly separated from one another [18-22]. Recent analysis of BAC clones supports this view [23]. In order to demonstrate trends of interest, we will assume a representative island size of 3,000 bases, but will additionally examine several hypothetical islands that are multiples of this value (Table 2).

**Table 2:** Island characteristics for an idealized 540 Mb filtered maize library

nominal island type	*σ*	*i*
1 gene	3,000	180,000
2 genes (hypothetical)	6,000	90,000
4 genes (hypothetical)	12,000	45,000

**Figure 2:**
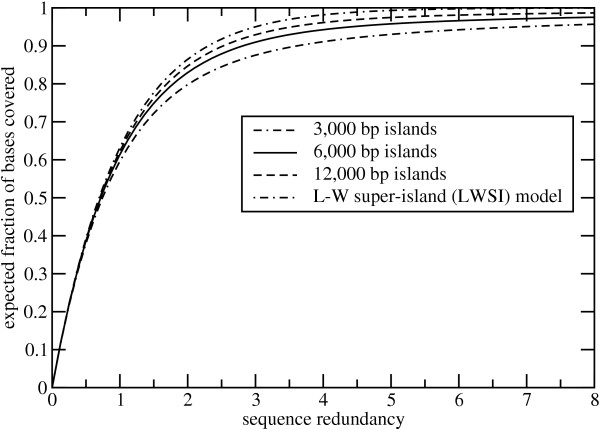
Coverage evolution for both the discontinuous island model and the LW "super-island" (LWSI) model.

Evolution of the coverage process is shown in Fig. 2 for the various island lengths, as well as the idealized LWSI model [11]. Evidently, there is little difference in performance up to about 1× sequence redundancy. That is, coverage is largely independent of the size of islands in the library. This reflects the tendency of reads to generate new coverage early in a project, rather than increasing overlaps of existing coverage. Gaps in the library appear to have little influence in this stage. Even in the case of small islands, it is likely that reads are preferentially populating the uncovered, central portions of the various islands.

As more reads are processed, we would expect this trend to change. Recall that the probability of a read covering a specific base position decreases closer to the edge of an island. This is the "edge effect". In this case, reads will tend to generate commensurately higher rates of overlap in the central regions, while the end regions will be covered at a slower pace. Indeed, Fig. 2 shows that behavior begins to diverge appreciably above 2× redundancy. Coverage becomes a strong function of island size.

Fig. 2 also indicates that the fraction of a filtered library that one can reasonably hope to obtain via random methods depends upon island size. For example, in the typical case of 3,000 bp islands, one would still expect to be missing about 4% of the sequence after processing 8× worth of reads. This figure contrasts with a 4% vacancy rate at slightly more than 3× redundancy with conventional libraries. Here, we would anticipate essentially complete coverage at the 8× milestone. For libraries consisting of 6,000 bp and 12,000 bp islands, the situation is more favorable. The model predicts vacancy rates of only about 2.5% and 1.3%, respectively, at 8× redundancy. Directed methods may be necessary for resolving the sequence at island edges.

The above observations call attention to a somewhat puzzling difference between filtered and conventional libraries. It is well-known that longer reads yield improved coverage performance for the latter. Specifically, coverage goes exponentially according to the redundancy, defined as *nλ/G*, where *G *is the genome size. Increasing the read length, in particular the ratio *λ*/G, implies that commensurately higher coverages could be obtained with a given number of reads. However, we have just observed that increasing the analogous ratio *λ/σ *in filtered libraries seems to slow the overall coverage rate.

This rather paradoxical behavior can be explained precisely in terms of the edge effect. In examining Thm. 3 more closely, we see that the first term (the one having a coefficient 2*i*) quantifies the coverage dynamics of the end regions. The coverage probability for any specific base in this region is not a function of read length (see Proof of Thm. 3), but the *fraction *of the island affected in this way is. Thus, longer reads impart edge effects over a larger percentage of each island. Moreover, the average difference in coverage probability between boundary and interior regions for a read is *λ*/2. Thus, the disparity in coverage probability between the two regions also grows in proportion to read length. Again paradoxically, this effect starts to diminish if reads become sufficiently long and finally vanishes in the limit of *λ *→ *σ*. However, this is simply a consequence of the fact that all base positions once again have an equal chance of being covered, so edge effects disappear. Although perhaps not obvious, this limiting case is described by Thm. 5.

### Gap census and contig length trends

**Figure 3:**
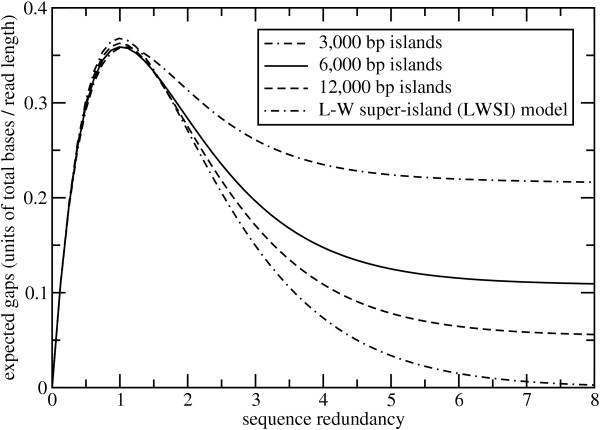
Evolution of gap census for both the discontinuous island model and the LWSI model.

Again using maize parameters as an example, Fig. 3 shows evolution of sequence gaps for the three island lengths in Table 2, as well as the idealized LWSI model. These curves are computed from Thm. 2 and are shown in the usual units of *i σ/λ *[10].

As with coverage, performance appears to be mostly independent of island size up to about 1× sequence redundancy, but the cases differ appreciably after that. The rates of gap closure decline significantly as the islands become smaller. Underlying dynamics are similar to those discussed above for coverage. It is worth noting that these trends are fundamentally different from what one realizes when varying effective read length. In that instance, the apparent number of gaps *rises *as reads become effectively shorter. We find a similar behavior here when island length is held fixed, although the convergence point is independent of read length (data not shown). This effect is a rather subtle consequence of the original method devised for modeling detection thresholds. It is discussed extensively in ref. [10], primarily in the context of fingerprint mapping. However, the phenomenon is not as relevant to shotgun sequencing because detection thresholds are small relative to read length and largely constant. Here, we expect island size to be the more influential variable.

Strictly speaking, library and sequence gaps are not completely independent of one another as we have implied here. For instance, the generation of a library gap is synonymous with placing a read in the end position of an island. This event may inadvertently eliminate a sequence gap, as well. We cannot rigorously claim that the total number of gaps at any given point is simply the sum of the two gap types. However, according to Thm. 1, the actual number of library gaps should always be small compared to the number of sequence gaps (data not shown). To be more specific, the rate of library gap formation is very slow; there are only *i *placements of a possible *i *(*σ *- *λ *+ 1) for which a read will spawn such a gap. Consequently, we can take the sequence gap census alone as a good approximation for the total number of gaps.

**Figure 4:**
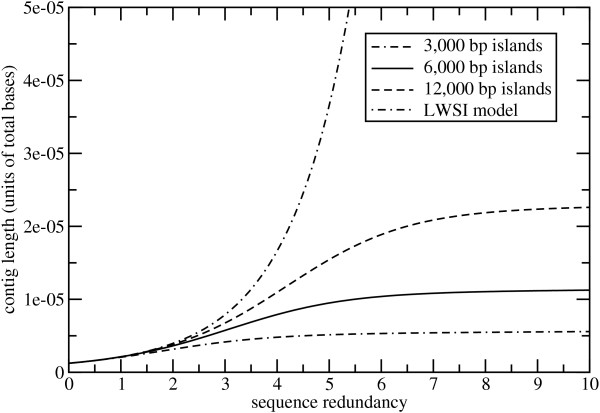
Evolution of average contig length for both the discontinuous island model and the LWSI model.

Assuming independence of the variables, we can approximate the expected length of contiguous segments as *E*〈*C*〉/*E*〈*S*〉. This expression is plotted in Fig. 4. Note that curves derived from the filtered model converge essentially to their respective island lengths, while the LWSI model diverges. This is a well-known anomaly in the basic Lander-Waterman formulation [24], although it has since been resolved [25]. Convergence to maximum contig length also appears to be faster for shorter islands. For example, for 3,000 bp islands there is little increase in average contig length after 5× sequence redundancy, while the 12,000 bp case is still developing even at 7× redundancy. Given the fundamental difference in longer-term behavior, it is somewhat surprising that the LWSI seems to be a better short-term indicator for contig length as compared to coverage and gaps. Specifically, predicted lengths seem to be independent of island size up to about 2× sequence redundancy, rather than the 1× limit observed for the other variables.

### Application for gene tagging

One of the growing applications we anticipate for filtered libraries is as a sampling method to rapidly prototype gene sets. This procedure is referred to as "gene tagging" [13,14]. Here, one simply obtains a light random sampling of the filtered library and assesses gene hits via homology searching. A number of fundamental questions revolve around how gene hits will be distributed for a given number of sequencing reads. If we take island hits as an analog of gene hits, Thms. 4 and 5 are useful for formulating predictions. Conversely, the LWSI model is not suited to such calculations because there is no consideration of how islands are actually separated from one another.

Investigators are often interested in rudimentary estimates of the number of genes hit, for which we can apply either of these theorems. Here, the governing parameter is exp(-*n/i*), so that island and read lengths are irrelevant. Data from two recent projects are available for comparison: a methyl-filtered sorghum (*Sorghum bicolor*) library sampled at roughly 1.1× redundancy [14] and a combination methyl-filtered high-Cot maize (*Zea mays*) library sampled at roughly 1.2× redundancy [13]. In the former case, library size and average gene size were estimated as *iσ *≈ 262 Mb and *σ *≈ 3 kb, respectively. Tagging results are based on comparisons to 137 genes annotated from finished sorghum BAC clones [14]. For the latter case, we calculate theoretical performance using the maize estimates described above. Maize tagging results are based on WU-BLASTN (W. Gish, personal communication) comparisons to 151 highly-annotated maize B73 genes [26] at a minimum identity of 98%.

**Figure 5:**
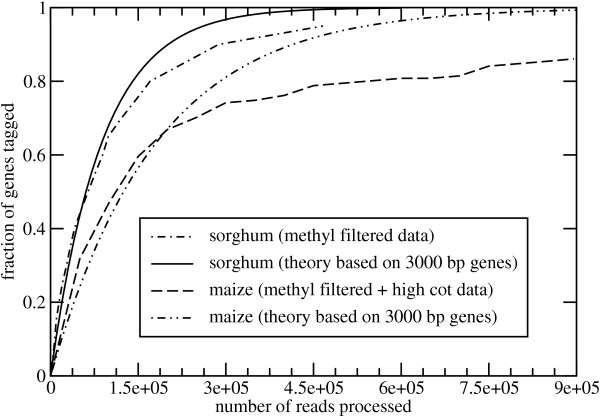
Comparison of Thm. 5 to experimental gene-tagging results in sorghum and maize.

Fig. 5 shows the expected fraction of genes hit according to both Thm. 5 and the experimental data. Theoretical curves depend on the number of islands, as calculated from parameter estimates. In particular, the sorghum library is modeled as having *i = *262 × 10^6^/3,000 = 87, 333 islands, while the number of maize islands is estimated at 540 × 10^6^/3,000 = 180,000. Agreement is relatively good in both cases up to about 60–70% of the gene space, after which the theory begins to over-predict the actual gene representation. Here, each empirical curve lies >20 standard deviations below its respective theoretical prediction (data not shown). This suggests systematic rather than stochastic factors account for the difference. Specifically, biases in the data are assumed to be present, although they are difficult to characterize at this stage. For example, Bedell et al. [14] speculate that perhaps 10% of sorghum repeats may be under-methylated, and thus able to survive the filtering process to some degree. Similarly, Whitelaw et al. [12] found a non-trivial number of retrotransposons in their combined methyl-filtered high-Cot maize library. Tagging also depends on the ability to identify suitable genes to assess, which itself is difficult and subject to error.

**Figure 6:**
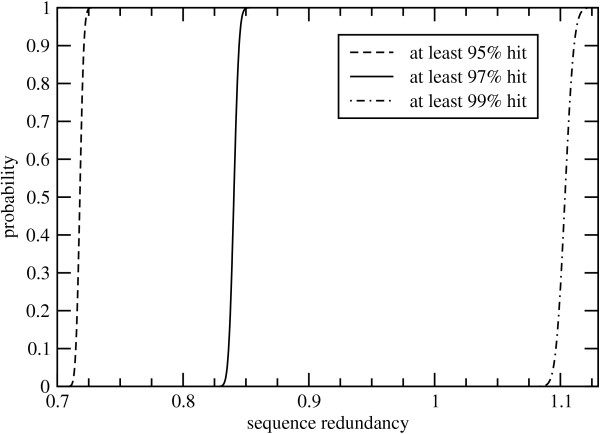
Tail probabilities for tagging various fractions of the gene space in *Sorghum bicolor *[14].

A more sophisticated calculation can be made with the probability distribution given by Thm. 5. Again using the parameters cited by Bedell et al. [14], we plot the tail probability of hitting various fractions of the gene space as a function of sequence redundancy in Fig. 6. As we would intuitively expect, the required redundancy increases with the fraction of the gene space desired. The curves are surprisingly sharp in all cases. That is, the theoretical milestones for gene-tagging appear to be very-well defined. For example, the probability of tagging at least 95% of the gene space is vanishingly small below 0.71× sequence redundancy, but approaches unity upon reaching 0.73× redundancy. These analyses are clearly subject to the biases discussed above. For example, Fig. 6 suggests that the 1.1× sequencing depth should probably have captured about 99% of the sorghum genes. However, Bedell et al. [14] calculated the actual value to be about 95%. The gene tagging process becomes more efficient as gene size increases because the number of genic islands is commensurately less for a given library size (data not shown).

### Estimating genic enrichment

Whitelaw et al. [12] proposed the idea of using the gap census predicted by LW theory, specifically the LWSI adaptation, to compute effective filtered genome size *G*_*L *_from preliminary shotgun data. The LW equation can readily be solved for *G*_*L *_as



so that the number of sequence gaps *E*〈*S*〉 serves as an indicator of *G*_*L*_. One can then estimate a genic enrichment factor *G/G*_*L*_, where *G *is the full genome size. Whitelaw et al. [12] performed such calculations for methyl-filtered and high-Cot maize libraries at less than 0.5× redundancy. Bedell et al. [14] also applied this concept to a methyl-filtered sorghum library at about 1× redundancy.

These calculations are founded on speculation that library gaps and edge effects could be ignored. We already described how performance is essentially independent of such factors when sequence redundancy remains below 1×. It therefore appears that these two particular computations are reasonable. However, this is clearly not the case in general. Standard LW theory will tend to under-estimate gaps, and consequently to under-estimate *G*_*L *_for higher redundancies. Genic enrichment factors would be artificially high. From a practical standpoint, light shotgun redundancy in conjunction with Eq. 1 seems to be a legitimate and convenient way to characterize enrichment; there is little penalty in neglecting edge effects and one need not estimate island size.

We note that *G*_*L *_in Eq. 1 can be further characterized in terms of lower and upper bounds using the appropriate distribution moments [25]. For example, Whitelaw et al. [12] calculated the size of the combination methyl-filtered high-Cot maize library to be roughly 413 Mb. Performing similar computations at 3 standard deviations above and below the mean, we estimate that the lower and upper limits for library size are approximately 406.6 and 420.5 Mb, respectively.

Peterson et al. [7] proposed a method for the complementary task; they compute the number of reads needed to cover a given filtered library fraction based on the "super-island" assumption inherent in the Clarke-Carbon equation [11]. According to the above discussion, this is, in principle, a reasonable approach. However, their specific calculations are synonymous with a redundancy exceeding 4× (99% coverage), making their estimates for *n *too low. In fact, Fig. 2 suggests that edge effects will make 99% random coverage difficult to achieve for any filtered library.

## Conclusion

The primary assumption associated with DNA processing models is that entities are distributed in an IID fashion. Because there is little in the broad spectrum of experimental data that corroborates this supposition [27], we must regard our results in the context of upper bounds of performance. Actual projects should generally fall somewhat short of predictions. Moreover, it can be difficult to *a priori *estimate input parameters, especially the number and average size of islands. Consequently, theoretical results for specific projects should be interpreted with these limitations in mind.

According to the trends discussed here, it is clear that if island size is sufficiently large compared to read length, the LWSI model will be sufficient for predicting a number of relevant parameters. However, with the exception of enrichment estimation discussed above, it does not appear that this will be the case for most projects. For example, we examined island sizes up to 12 kb, but edge effects were still noticeable for reads ~700 bp in length. It is unclear whether there are species whose average island length would be substantially larger than this. Moreover, there is an ongoing trend toward longer reads [28]. Coupled with the need to calculate island-specific parameters, we feel the model described here will play a role for filtered library projects analogous to the one already established by standard LW theory for conventional libraries.

We also suggest potential application of this model for other non-traditional sequencing scenarios. For example, there is increasing interest in sequencing ciliated protozoa [29,30]. The macronuclear genomes of such organisms consist of >20,000 distinct "nano-chromosomes", with an average length of less than 3,000 base pairs. Because most of these chromosome structures are too long to be traversed with end-sequences, it is likely that a shotgun approach will be necessary.

The observations made here have a number of practical implications for the planning and execution of future filtered library shotgun projects. In general, the progress realized when using standard "full-length" reads will be less than that of the equivalent WGS project. In many cases, this implies stopping at what are conventionally considered to be only moderate redundancies. For example, results shown in Figs 2, 3, and 4 suggest little is gained in sequencing 3 kb islands past about 5×. Likewise, they indicate that assemblies would have less sequence coverage and less contiguity as compared to equivalent WGS projects. Improved economy and performance of directed methods become commensurately more important.

The model establishes *λ/σ *as the primary parameter governing edge effects. By varying island size, we found that results for a given value of sequence depth improved as *λ/σ *decreases. The same effect can clearly be obtained by decreasing read length for a given island size. Pyro-sequencing platforms immediately suggest themselves as a good potential match for this application. For example, current effective read lengths of about 150 bp [31] imply *λ/σ *= 0.05 for 3 kb islands. Here, results would be roughly equivalent to what is shown for the 12 kb islands in Figs 2, 3, and 4 using full-length reads. That is, contiguity and sequence coverage would be much improved. Because islands correlate with low-copy sequence content, we would not expect reduced read lengths to substantially impede the assembly process.

## Methods

**Figure 7:**
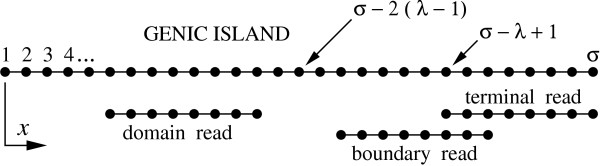
Island coordinate system and nomenclature.

This section describes the mathematical proofs for the theorems reported in the Results section. First, we define a nucleotide-based island coordinate system *x *∈ {1, 2, 3, ..., *σ*} whose origin is the left-hand boundary (Fig. 7). Coordinate locations for sequencing reads refer to the starting location of the read, i.e. the position of its left-most base. Each read falls into one of three classifications.

1. *Domain Read: *A read for which no overlapping read will extend past the right end of the island. The coordinate range is *x *∈ {1, 2, 3,..., *σ *- 2(*λ *- 1)}.

2. *Boundary Read: *A read for which one or more overlapping reads can extend past the right boundary. The coordinate range is *x *∈ {*σ *- 2(*λ *- 1) + 1,..., *σ *- *λ*}.

3. *Terminal Read: *A read that resides on the extreme right of the island. Its position is *x *= *σ *- *λ *+ 1.

There are no read starting positions for *x *> *σ *- *λ *+ 1. In LW theory, all reads are of the domain type because the target sequence is considered infinitely long. The additional concepts of boundary and terminal reads allow us to account for library gaps and edge effects in a structured fashion.

### Proof of theorem 1

A library gap on an island is manifested by the presence of a terminal read, which can be placed in exactly one way. By Lemma 1, the associated probability is *ξ *(1). Considering this event over all *i *islands in conjunction with the IID assumption yields theorem 1.

### Proof of theorem 2

This proof is based on the presumption 2*λ *≤ *σ *+ 1, which we anticipate would characterize most library filtering projects. That is, islands are long enough such that domain reads actually exist. For shorter islands, appropriate results can be derived by simply omitting the consideration of domain reads.

Let us define event *Θ*_*x *_as the start of a contig of reads at position *x *on an island, where *x *is constrained according to the above definitions of the read types. Also, define the sub-events , whereby the contig is initiated by reads 1, 2,..., *n*, respectively. Now, , so that



As in LW theory, we may consider the sub-events to be mutually-exclusive of each other (Lemma 2), from which we find



This expression represents the probability that a contig starts at position *x *on an island. For cases of biological interest, *n *≪ Π, as described in Lemma 2. If this condition is not met, one must instead utilize the full binomial placement model as discussed in Lemmas 1 and 2.

Following Lander and Waterman [10], we observe that a contig begins with the initiation of a "base read" at *x *and continues until no overlapping reads are detected. This event is denoted by Φ_*x *_and can be taken to represent a gap associated with position *x *on the island. For domain reads of the type considered in LW theory, overlapping reads can be found along the entirety of the base read. Here,



where the overbar represents the complement of the specified event. In light of the IID assumption, the corresponding probability is



For boundary reads, the number of possible overlapping reads depends explicitly on the distance of the base read from the end of the island. Analysis reveals a similar expression, except where the power *λ *in the previous expression is replaced by *σ *- *λ *+ 2 - *x*.

Asymptotic approximation can be applied to *P *(Φ_*x*_) for domain reads according to the argument discussed in Lemma 2 for *λ *and the ratio *n*Π^-1^. However, it is not valid for boundary reads because *σ *- *λ *+ 2 - *x *is not sufficiently large, in general. Considering *P *(Φ_*x*_) for every domain or boundary read position *x *on each one of the *i *islands in conjunction with the IID assumption then yields theorem 2.

### Proof of theorem 3

This proof is based on the presumption 2*λ *≤ *σ*, which we anticipate would characterize most library filtering projects. That is, there are no base positions that would be covered with complete certainty for any read that hits the island. For shorter islands, appropriate results can be derived in a similar fashion to that shown here.

According to Lemma 1, the probability *P *(Θ_*x*_) of a clone traversing a specific position *x *on an island can be deduced by simply counting the number of ways a segment can cover this position. If *x *<*λ*, the left boundary constrains the position, giving exactly *x *successful placements. Likewise, symmetry dictates the same behavior at the right boundary, so that the number of placements is *σ *- *x *+ 1 when *x *> *π*. For each of the remaining positions, no boundary constraints exist, so that there are *λ *successful placements. Therefore,



where again, function *ξ *is defined by Lemma 1. Considering *P *(Θ_*x*_) for every position *x *on each one of the *i *islands in conjunction with the IID assumption then yields theorem 3.

For segment lengths *λ *> *σ*/2, probability is identical to the above for the first two cases, but their limits are changed to *x *<*π *and *x *> *λ*, respectively, and the last case becomes *ξ *(*π*). Expected value of coverage could then be found by similar algebraic operations.

### Proof of theorem 4

The number of reads placed on an island does not depend upon position. Each read either hits a specific island with probability *p *= *i*^-1^, or it does not hit this island with  = 1 - *i*^-1^. The process is binomial since the *n *reads are IID. However, as *i *and *n *are both large for cases of interest, it essentially behaves according to a Poisson distribution having a rate *n/i*.

### Proof of theorem 5

By Thm. 4, the probability that an island is hit by at least one read is 1 - exp(-*n/i*). The expected number of islands hit is obtained by considering this case for all *i *islands. Since position is irrelevant, this process is equivalent to the classical occupancy problem having *i *bins. Feller [32] reports the asymptotic distribution.

### Supporting lemmas

The following Lemmas represent simplifications of binomial processes that are valid for typical filtered genomic libraries, i.e. those having many islands. Scenarios in which the number of islands is small can readily revert to the underlying binomial descriptions.

**Lemma 1. ***The probability that a particular event occurs on a specific island is*

*ξ *(*β*) = 1 - *e*^-*β n*/Π^,

*where β denotes the number of local read placements on the island associated with realizing the event*.

*Proof*. Let Φ_*j *_denote realization of the event on the island in question for a specific read *j*. We have Φ_*j *_= Θ_1 _∩ Θ_2_, where Θ _1 _and Θ_2 _denote, respectively, that read *j *lands on the required island and that it instantiates the event. All islands are identical, so Θ_2 _does not depend on Θ_1_. If there are *β *placements that instantiate the event on an island, the probability is



which can be written more succinctly as *P *(Φ_*j*_) = *β*/Π. The probability of not realizing the event for a specific read *j *is simply the complement .

Reads are of uniform length and are independent of one another, thus satisfying the IID assumption. Consequently, this scenario is binomial over the collection of *n *reads; each read either instantiates the event, or it does not. The probability of not realizing the event for any of the *n *reads is clearly



The probability of the main event itself is simply the probability that it is caused by one or more reads: .

To complete the proof, we must show that the asymptotic form is valid. The relevant functions expand as



and



which are clearly equivalent in an asymptotic sense as *n *becomes large. In the limit *β *→ *σ *- *λ *+ 1, all placements on an island instantiate the event, so that *β*/Π → *i*^-1^. This represents the worst case for the approximation's accuracy. For the typical filtered library, we expect *i *> 10^4^, so that the exponential form would be valid for characteristic values of *n*. For example, after *n *= 10^4 ^reads the approximation error would be limited to a maximum value of about 0.005%.

**Lemma 2. ***The start of a contig can be considered according to a mutually-exclusive read placement process having a probability n*/Π.

*Proof*. Let  denote the event where read *j *starts at position *x *and thereby instantiates a contig at that location. The probability of this event is clearly binomial; the read either starts a contig at *x *with probability 1/Π, or it does not with complementary probability 1 - 1/Π. The probability of at least one of the *n *reads starting a contig is given by Lemma 1 as *ξ *(1). This quantity expands as



Let us define *ζ *= *λ*/*σ*, so that the quotient *n*/Π can be recast as



Here, Δ = *nλ *(*iσ*)^-1 ^is the conventional sequence redundancy, which is usually less than 10. Read lengths are typically *λ *> 500, while we anticipate *ζ *< 0.25 for most biologically-relevant cases. Also, *σ*^-1 ^≪ 1, making its contribution negligible. Consequently, *n*/Π is, at most, on the order of about 0.02. This implies the above expansion is well-approximated by its first term alone, with the maximum error being about 1% for the stated parameters. This one-term approximation is identical to what one obtains from a strict model of a mutually-exclusive starting process, i.e.



## Authors' contributions

MCW and WBB conceived the model. MCW performed the mathematical derivation of the model and drafted the manuscript. WBB obtained and analyzed the maize comparison data and edited the draft manuscript. Both authors read and approved the final manuscript.

## References

[B1] Tettelin H, Nelson KE, Paulsen IT, Eisen JA, Read TD, Peterson S, Heidelberg J, DeBoy RT, Haft DH, Dodson RJ, Durkin AS, Gwinn M, Kolonay JF, Nelson WC, Peterson JD, Umayam LA, White O, Salzberg SL, Lewis MR, Radune D, Holtzapple E, Khouri H, Wolf AM, Utterback TR, Hansen CL, McDonald LA, Feldblyum TV, Angiuoli S, Dickinson T, Hickey EK, Holt IE, Loftus BJ, Yang F, Smith HO, Venter JC, Dougherty BA, Morrison DA, Hollingshead SK, Fraser CM (2001). Complete Genome Sequence of a Virulent Isolate of *Streptococcus pneumoniae*. Science.

[B2] International Human Genome Sequencing Consortium (2004). Finishing the Euchromatic Sequence of the Human Genome. Nature.

[B3] SanMiguel P, Tikhonov A, Jin YK, Motchoulskaia N, Zakharov D, Melake-Berhan A, Springer PS, Edwards KJ, Lee M, Avramova Z, Bennetzen JL (1996). Nested Retrotransposons in the Intergenic Regions of the Maize Genome. Science.

[B4] Palmer LE, Rabinowicz PD, O'Shaughnessy AL, Balija VS, Nascimento LU, Dike S, de la Bastide M, Martienssen RA, McCombie WR (2003). Maize Genome Sequencing by Methylation Filtration. Science.

[B5] Bennetzen JL, Chandler VL, Schnable P (2001). National Science Foundation-Sponsored Workshop Report. Maize Genome Sequencing Project. Plant Physiology.

[B6] Rabinowicz PD, Schutz K, Dedhia N, Yordan C, Parnell LD, Stein L, McCombie WR, Martienssen RA (1999). Differential Methylation of Genes and Retrotransposons Facilitates Shotgun Sequencing of the Maize Genome. Nature Genetics.

[B7] Peterson DG, Schulze SR, Sciara EB, Lee SA, Bowers JE, Nagel A, Jiang N, Tibbitts DC, Wessler SR, Paterson AH (2002). Integration of Cot Analysis, DNA Cloning, and High – Throughput Sequencing Facilitates Genome Characterization and Gene Discovery. Genome Research.

[B8] Yuan Y, SanMiguel PJ, Bennetzen JL (2003). High-Cot Sequence Analysis of the Maize Genome. Plant Journal.

[B9] Rabinowicz PD, Palmer LE, May BP, Hemann MT, Lowe SW, McCombie WR, Martienssen RA (2003). Genes and Transposons are Differentially Methylated in Plants, but not in Mammals. Genome Research.

[B10] Lander ES, Waterman MS (1988). Genomic Mapping by Fingerprinting Random Clones: A Mathematical Analysis. Genomics.

[B11] Clarke L, Carbon J (1976). A Colony Bank Containing Synthetic Col El Hybrid Plasmids Representative of the Entire E. coli Genome. Cell.

[B12] Whitelaw CA, Barbazuk WB, Pertea G, Chan AP, Cheung F, Lee Y, Zheng L, van Heeringen S, Karamycheva S, Bennetzen JL, SanMiguel P, Lakey N, Bedell J, Yuan Y, Budiman MA, Resnick A, van Aken S, Utterback T, Riedmuller S, Williams M, Feldblyum T, Schubert K, Beachy R, Fraser CM, Quackenbush J (2003). Enrichment of Gene-Coding Sequences in Maize by Genome Filtration. Science.

[B13] Springer NM, Xu XQ, Barbazuk WB (2004). Utility of Different Gene Enrichment Approaches Toward Identifying and Sequencing the Maize Gene Space. Plant Physiology.

[B14] Bedell JA, Budiman MA, Nunberg A, Citek RW, Robbins D, Jones J, Flick E, Rohlfing T, Fries J, Bradford K, McMenamy J, Smith M, Holeman H, Roe BA, Wiley G, Korf IF, Rabinowicz PD, Lakey N, McCombie WR, Jeddeloh JA, Martienssen RA (2005). Sorghum Genome Sequencing by Methylation Filtration. PLOS Biology.

[B15] Meyers BC, Tingey SV, Morgante M (2001). Abundance, Distribution, and Transcriptional Activity of Repetitive Elements in the Maize Genome. Genome Research.

[B16] Bennetzen JL, Schrick K, Springer PS, Brown WE, SanMiguel P (1994). Active Maize Genes are Unmodified and Flanked by Diverse Classes of Modified, Highly Repetitive DNA. Genome.

[B17] Martienssen RA, Rabinowicz PD, O'Shaughnessy A, McCombie WR (2004). Sequencing the Maize Genome. Current Opinion in Plant Biology.

[B18] Tikhonov AP, SanMiguel PJ, Nakajima Y, Gorenstein NM, Bennetzen JL, Avramova Z (1999). Colinearity and its Exceptions in Orthologous adh Regions of Maize and Sorghum. Proceedings of the National Academy of Sciences.

[B19] Fu H, Dooner HK (2002). Intraspecific Violation of Genetic Colinearity and its Implications in Maize. Proc Nat Acad Sci.

[B20] Song RT, Messing J (2002). Contiguous Genomic DNA Sequence Comprising the 19-kD zein Gene Family from Maize. Plant Physiology.

[B21] Ilic K, SanMiguel PJ, Bennetzen JL (2003). A Complex History of Rearrangement in an Orthologous Region of the Maize, Sorghum, and Rice Genomes. Proceedings of the National Academy of Sciences.

[B22] Langham RJ, Walsh J, Dunn M, Ko C, Goff SA, Freeling M (2004). Genomic Duplication, Fractionation and the Origin of Regulatory Novelty. Genetics.

[B23] Danforth Center Maize Clone Viewer. http://maizeapache.ddpsc.org/cgi-bin/gbrowse.cgi?source=03_jb_genes.

[B24] Roach JC (1995). Random Subcloning. Genome Research.

[B25] Wendl MC, Waterston RH (2002). Generalized Gap Model for Bacterial Artificial Chromosome Clone Fingerprint Mapping and Shotgun Sequencing. Genome Research.

[B26] TIGR Bac Annotations. http://www.tigr.org/tdb/tgi/maize/bac_annot.shtml.

[B27] Wendl MC, Yang SP (2004). Gap Statistics for Whole Genome Shotgun DNA Sequencing Projects. Bioinformatics.

[B28] Elkin C, Kapur H, Smith T, Humphries D, Pollard M, Hammon N, Hawkins T (2002). Magnetic Bead Purification of Labeled DNA Fragments for High-Throughput Capillary Electrophoresis Sequencing. Biotechniques.

[B29] Prescott DM, Prescott JD, Prescott RM (2002). Coding Properties of Macronuclear DNA Molecules in *Sterkiella nova (Oxytricha nova)*. Protist.

[B30] Doak TG, Cavalcanti ARO, Stover NA, Dunn DM, Weiss R, Herrick G, Landweber LF (2003). Sequencing the *Oxytricha trifallax *Macronuclear Genome: A Pilot Project. Trends in Genetics.

[B31] Ronaghi M (2001). Pyrosequencing Sheds Light on DNA Sequencing. Genome Research.

[B32] Feller W (1968). An Introduction to Probability Theory and Its Applications.

